# Development and field testing of a patient decision aid for management of acute Achilles tendon rupture: a study protocol

**DOI:** 10.1186/s12911-021-01589-5

**Published:** 2021-07-24

**Authors:** Brad Meulenkamp, Julia Brillinger, Dean Fergusson, Dawn Stacey, Ian D. Graham

**Affiliations:** 1 Department of Medicine, University of Ottawa, Ottawa, Canada; 2 Ottawa Hospital Research Institute, Ottawa, Canada; 3 Centre for Practice-Changing Research, Ottawa Hospital Research Institute, Ottawa, Canada; 4 Faculty of Health Sciences, University of Ottawa, Ottawa, Canada; 5 School of Epidemiology and Public Health, University of Ottawa, Ottawa, Canada; 6 Orthopaedic Trauma, Foot and Ankle Surgery, The Ottawa Hospital, Ottawa, Canada

**Keywords:** Achilles tendon rupture, Decision aid, Shared decision-making

## Abstract

**Background:**

Achilles tendon ruptures are common injuries in an otherwise healthy, active population. Several treatment options exist, with both surgical and non-surgical options. Each treatment option has a unique set of risks and harms, which may present patients with decisional conflict. The aim of the proposed study is to develop, alpha test and field test a patient decision aid for patients presenting with acute Achilles tendon ruptures.

**Methods:**

This is a three-stage study protocol. First, we will assemble a multi-disciplinary steering group including patients, clinicians, educators, and researchers to develop the patient decision aid prototype using the Ottawa Decision Support Framework. Second, we will perform a mixed-methods alpha test of the decision aid prototype with patients and clinicians experienced in acute Achilles tendon ruptures. Outcomes measured will include acceptability and usability of the patient decision aid measured using validated outcome scales and semi-structured interviews. A minimum of three rounds of feedback will be obtained. Results will be analyzed using descriptive statistics, reviewed by the steering group, to guide revisions to decision aid prototype at each round. The third stage will be field testing the revised decision aid prototype in usual clinical care. A pre-/post-study will be performed with patients with acute Achilles tendon ruptures. Patients will be recruited from the emergency department and complete the pre-consultation decision aid prior to a one-week follow up with their surgeon. The primary outcome of field testing will be feasibility of implementing the decision aid in the clinical setting and will be measured with recruitment and completion metrics. Secondary outcomes include acceptability of the decision aid, knowledge, preparedness for decision making, and decisional conflict, measured using validated outcome measures. Statistical analysis will be performed using descriptive analysis for primary outcomes and a student t-test and Wilcoxon Rank-Sum test for secondary outcomes.

**Discussion:**

This comprehensive study protocol outlines the development, alpha testing, and field testing of a patient decision aid for patients with acute Achilles tendon rupture. Systematic and transparent development and testing of patient decision aids is critical to improve decision aid quality.

*Trial registration* Not Applicable.

**Supplementary Information:**

The online version contains supplementary material available at 10.1186/s12911-021-01589-5.

## Background

Patients presenting to hospital with an acute Achilles tendon rupture are faced with an important decision regarding treatment management: to have surgery or pursue conservative management. There are harms and benefits to each approach. Historically, non-operative care has been associated with a higher tendon re-rupture rate, and surgery with the risks of surgical complications including infections, wound healing problems, and subsequent surgery [1].

The decision is made more complex by the existence of varying non-operative and surgical treatment options [1, 2]. Non-operative management has evolved from a period of prolonged immobilization in a cast to allow tendon healing, to early motion and functional rehabilitation protocols. Use of these protocols has led to decreased re-rupture rates, but requires early patient enrollment, high patient engagement, and ideally, access to physiotherapy to provide optimal outcomes [1]. Surgical care is also evolving, from traditional open surgery, to more percutaneous and minimally invasive options that attempt to decrease wound healing problems. These surgical techniques, however, come with increased risk of other harms, including nerve injury [3]. Given the evolution in the benefit-to-harm ratio, practice trends in Scandinavia [4] and Canada [5] have shifted towards non-operative functional rehabilitation, with rates of surgery declining.

When several reasonable treatment options exist with varying harms and benefits, patients often experience decisional conflict [6]. Decisional conflict is uncertainty over a course of action, and may result in worry, questioning of personal values, physical stress and ultimately, decision delay [7].

Meanwhile, the delivery of patient care is in the midst of a paradigm shift [8]. The patient-clinician relationship is changing from a paternalistic one of unilateral discussion to more of a patient-centered approach. The concept of shared decision-making (SDM) is the crux of this approach [9, 10]. SDM involves an exchange of information around health-care decisions, supplementing the patient-clinician discussion and allowing patients to make a more personal, values-based decision [11]. Decision support tools facilitate this process [11, 12].

Patient Decision-Aids (PtDAs) are tools that may be used by patients either in preparation for or within a consultation with their physician. They explicitly state the decision to be made and provide patient-friendly information on decision options, harms, and benefits in a format that allows the patient to clarify what matters to them most [13].

The utility of PtDAs has been widely studied with demonstrated effectiveness in improving patient knowledge, decreasing decisional conflict, and increasing patient participation in decision-making [13]. As facilitators of SDM, PtDAs may also lead to improved satisfaction with their patient experience [14].

There are no known PtDAs in the published literature to assist patients making treatment decisions regarding acute Achilles tendon ruptures. The aim of this study protocol is to develop and test a PtDA to help patients make a more informed, values-based decision when considering treatment options for acute Achilles tendon rupture.

## Methods

### Objectives

The specific objectives of this study are (a) to develop a PtDA for patients to use in preparation for the consultation; and (b) to field test the PtDA with patients and clinicians making involved in decisions regarding this treatment.

### Design

This study will involve a three-stage study protocol. First, we will assemble a multi-disciplinary steering group including patients, clinicians, educators, and researchers to develop the patient decision aid prototype using the Ottawa Decision Support Framework (ODSF). Second, we will perform a mixed-methods alpha test of the decision aid prototype with patients and clinicians experienced in acute Achilles tendon ruptures. Third, we will field test the revised decision aid prototype in a usual clinical care setting.

### Stage 1: developing the patient decision aid prototype

#### Guiding conceptual frameworks

The PtDA will be developed using the Ottawa Decision Support Framework (ODSF) [15] and in accordance with the International Patient Decision Aids Standards (IPDAS) quality criteria [16]. The ODSF is a theory-based model for helping guide patients making health decisions. It is grounded on cognitive, socioeconomic, and psychology theories and has been used in the creation dozens of PtDAs [15, 17], twenty-four of which have been evaluated in randomized controlled trials [17]. The IPDAS criteria were developed to systematically guide the PtDA development, content and evaluation, with agreement from over 100 stakeholders including patients, policy makers, clinicians and researchers [18].

#### Development of the patient decision aid

**Fig. 1:**
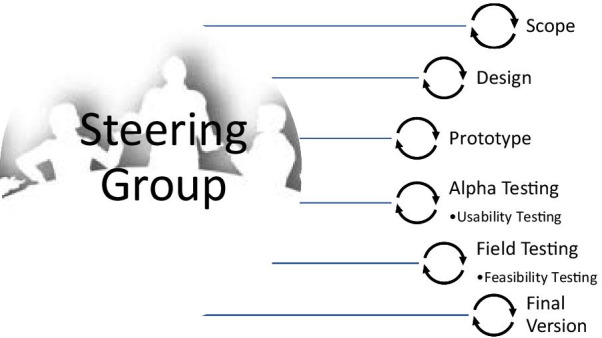
The IPDAS development model for patient decision aids

The IPDAS criteria specific to development have subsequently been updated and expanded to include steps to help guide the PtDA development process [19]. These steps include: (1) defining the purpose, scope and audience of the decision aid, (2) collecting and synthesizing the data for inclusion, (3) developing the PtDA prototype, (4) alpha testing by end-users to ensure usability of the PtDA, (5) field testing feasibility with end-users in the clinical setting, and (6) producing the final version (Fig. 1) [19].

#### Steering group

In keeping with an integrated knowledge translation (iKT) approach [20] and as recommended by the IPDAS [19], a steering group will be assembled to guide the PtDA development process. Central to the iKT approach is the involvement of and feedback from end-users longitudinally throughout the research process [20]. This approach has demonstrated benefits in terms of development of a more useful tool with increased end-user knowledge buy-in, uptake and impact [21–25]. For this protocol and subsequent study, the knowledge end-users include patients, physiotherapists, and orthopaedic surgeons. The steering group will be inter-disciplinary and will include end-users with content expertise, two members with methodological expertise in shared decision-making and PtDAs, patient educators, and a web-developer to facilitate creation of a web-based tool.

#### Establishing the scope of the PtDA

According to IPDAS, the scope of the PtDA involves establishing the specific decision, the target audience, and the purpose of the PtDA [19]. The purpose of this pre-consultation PtDA is to inform patients with acute Achilles tendon rupture about treatment options, as well as harms and benefits of each of the options. The goal is to assist patients in deciding on a course of treatment. Specifically, the decision to be made is “Should I have surgery or non-operative management for my Achilles tendon rupture?”.

#### Design of the PtDA

The steps in designing the PtDA prototype include an assessment of decisional needs of patients and clinicians, formulating a format and distribution plan, and reviewing and synthesizing the evidence for inclusion as content [19]. Recent literature demonstrates conflicting clinician opinions on how to advise patients with an Achilles tendon rupture [26, 27], and the first steering group agenda item will be a discussion with clinician and patient members to identify patients’ decisional needs. The steering group will develop and revise the PtDA through an iterative process until consensus is reached on content and structure. The prototype design will be based on the ODSF template [28] and it meets the IPDAS minimal development standards recommended for minimizing risk of bias in PtDAs [16].

The initial prototype will paper-based and drafted in English, with plans for final distribution electronically and printable paper versions available in clinical practice in both English and French translations. Treatment initiation for Achilles tendon rupture is time sensitive thus it is essential that the PtDA be administered to patients soon following injury. With accessibility being a key aspect of PtDA usability, offering both paper and electronic formats will facilitate and maximize patient uptake [29]. The PtDA will be designed knowing that the most common points of first patient contact is the emergency room or outpatient orthopaedic clinic.

The prototype will be written in plain language appropriate for an 8th grade reading level or lower as determined by the Flesh-Kincaid Readability Test Tool [30]. It will be formatted to include: (a) information on Achilles tendon ruptures and specify which patients are eligible for the PtDA, (b) treatment options to consider, (c) summarized evidence on benefits and harms of each approach, (d) a values clarification exercise, (e) space to indicate a preferred treatment option; and (f) the SURE test [31]. The SURE test is a 4-item questionnaire validated for screening for decisional conflict. Probabilities will be presented numerically and in words, with links to graphical representations to facilitate patients’ understanding and adhering to the IPDAS criteria specific to presenting probabilities [32]. There will also be links included for additional information about Achilles tendon ruptures, literature used to develop the PtDA, and treatment specifics including rehabilitation protocols.

### Stage 2: alpha testing

Alpha testing aims to evaluate the acceptability and usability of the decision tool from the perspective of patients and clinicians [19]. Alpha testing will be performed exclusively with the English version of the PtDA prototype. The steering group will discuss results and feedback from the alpha testing in an iterative fashion until consensus is reached on required revisions to the PtDA prototype and field testing outcomes of interest.

A mixed-methods study will be performed with both acceptability and usability questionnaires (Additional file 1: Appendix A, Additional file 2: Appendix B, Additional file 3: Appendix C), as well as qualitative descriptive methods used to optimize stakeholder feedback [33, 34]. This approach has the advantage of allowing evaluation of the outcomes of interest, while also allowing for participants to go beyond explicit questioning, expressing their thoughts and perspectives on the PtDA. This type of qualitative data collection and analysis allows ‘low inference’ discussion and analysis of participant’s responses without manipulation. Specifically, the primary data collection methods will be semi-structured interviews (SSI) with patients and clinicians, allowing for participants to elaborate and clarify responses.

#### Participants

Participants for alpha testing will be end-users of PtDA: clinicians and patients who have previously made this decision.

Clinician participants will include emergency room physicians, orthopaedic surgeons, and physiotherapists, all members of the care team typically involved in the diagnosis and care of these patients. Convenience sampling [35, 36] will be used to recruit clinician participants through professional networks. Members of the Division of Orthopaedic Surgery and Department of Emergency Medicine at the Ottawa Hospital, in addition to physiotherapists from the Riverside Hospital (Ottawa, Ontario) will be invited by email to participate in the study, as will orthopaedic surgeons from across Canada (through the Canadian Orthopaedic Foot and Ankle Society member email list). This will increase the diversity of participants and raise awareness of the PtDA for future dissemination. We will attempt to include both male and female surgeons to maintain diversity of the sample. Recruitment emails will include an information sheet explaining the purpose of the PtDA and the alpha testing.

A convenience sample of patients who have been or are actively being treated for an acute, first-time Achilles tendon rupture at TOH will be recruited for participation. Although this is predominantly a male compared to female injury (6:1) [2], we will strive to include both men and women patients that have been treated both with and without surgery. A study poster will be placed both in the orthopaedic clinic waiting area and eligible patients will be approached by a member of the clinical team to provide a description of the purpose and structure of alpha testing for the PtDA. Patients will be excluded if they are unable to understand the PtDA due to language barrier or visual impairment.

The target sample size for alpha testing will be 20 participants, with 10 patients and 10 clinicians. This is derived from the work of Faulkner [37], who demonstrated that problem identification was substantially decreased in usability testing with increasing participant size. With participant sizes of five, 10 and 20, the lowest percentage of problem identification increased from 55 to 80% and finally 95%, respectively [37].

#### Procedure

**Fig. 2:**
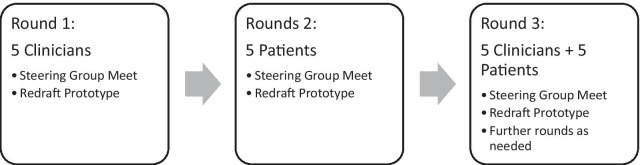
Alpha testing process

Alpha testing will be an iterative process of revising and redrafting the PtDA [19] using a minimum of three rounds with five participants in each round (Fig. 2). Further rounds will be added at the discretion of the steering group based on ongoing feedback from participants.

The first round of testing will be with five clinician participants who are routinely part of the care team of Achilles tendon rupture patients. This will include three orthopaedic surgeons, an emergency medicine physician, and a physiotherapist. Those who agree to participate will be sent a study package with consent form, a copy of the updated PtDA prototype, and alpha testing clinician outcome questionnaires. They will be contacted by the research assistant either in person or on the phone depending on geographic location and preference. Recruited clinician participants will be sent a $20 gift voucher. Following an explanation of the purpose of the study, participants will be asked to sign consent for participation. They will then review the PtDA, complete the outcome instruments and participate in the SSI with the research assistant. The steering group will meet to review outcome measures and interview feedback and decide on revisions to the PtDA prototype.

The second round of testing will be with five patients recruited from the Ottawa Hospital Foot and Ankle clinic. Patients will meet with the research assistant who will review the study goals and provide consent. To simulate the intended setting of the PtDA, they will take the PtDA home with instructions to review it and complete the alpha test patient outcome questionnaires. They will then be invited for an SSI either on the phone or in person depending on their comfort and preferences. Recruited patients will be given a $20 gift voucher for compensation for their time as well as a hospital-parking pass. Once again, results and feedback will be brought to the steering group for review and revisions to the prototype made as needed. Despite being a predominant male injury [2], we will aim for two female participants for each block of five so as not to miss potential important gender differences.

The third round of testing will be concurrently performed with five additional clinicians and five additional patients. The structure will remain the same as in previous rounds. If the steering group deems further prototype revisions necessary, subsequent recruitment will be performed in blocks of 3–5 participants.

#### Outcomes and instruments

Baseline clinician and patient demographics will be collected. For clinicians this will include profession, sex, gender, years in practice, number of Achilles tendon rupture patients treated monthly, and clinical subspecialty training for surgeons. Baseline patient demographics collected will include age, sex, gender, level of education, occupation and treatment status.

The primary outcomes of interest are acceptability and usability of the PtDA, which will be evaluated quantitatively and qualitatively. For measuring PtDA acceptability with both patients and clinicians, we will adapt the Ottawa Acceptability Tool (OAT) from the ODSF website [28]. For patients, this questionnaire (Additional file 1: Appendix A) evaluates user perception of the PtDA using a combination of closed and open-ended questions, specifically asking about length, perceived usefulness for decision support, amount of and balance of information presented. This tool has strong face validity and has been used extensively in PtDA evaluation with a wide range of conditions [38–40]. The OAT has also been adapted for use with clinicians with a 15-question format (Additional file 2: Appendix B) and will be used as the primary quantitative measure of acceptability for clinician participants.

Usability will be evaluated both quantitatively using the System Usability Scale (SUS) [41, 42] and qualitatively through feedback from the semi-structured interviews. The SUS is a validated [43] questionnaire (Additional file 3: Appendix C) using five-point Likert scale response categories, with a score over 68 indicating higher than average usability. It has been previously used in usability testing of PtDAs [44].

#### Semi-structured interviews

Semi-structured interviews will be conducted with separate interview guides for clinician and patient participants. Interview guides will be reviewed by the steering group. For clinicians, a series of open and closed-ended questions will be asked about process usability, mode of delivery in the clinical setting, and feedback on content (length, language, information and balance). For patients, the focus of the interview will be on presentation (layout and format) and content (length, language, information and balance) to help ensure the end product is optimized for the end user. Interviews will be recorded and transcribed.

#### Data analysis

Data will be analyzed iteratively following each round of testing. Quantitative data will be coded and stored in an encrypted Microsoft Excel spreadsheet. Descriptive statistics will be used to summarize data from the OAT and SUS. Response frequencies from the patient OAT will be reported and dichotomized into positive and negative responses. Given the small sample size, we will use medians and ranges for the practitioner OAT and SUS. Qualitative data, including feedback from the OAT and SSIs will be compiled and analyzed using thematic analysis [45, 46]. This will be performed in 6-phases, beginning with (1) transcription and initial data review, (2) broad generation of initial codes, (3) collating codes into themes, (4) reviewing the themes to either further collapse or expansion, (5) defining and naming the themes and (6) generating a final report.

The steering group will perform iterative data review following each round of testing. Revisions will be discussed and suggested when negative responses are encountered on the patient OAT or SUS, median scores less than three on the clinician OAT when further suggestions are found through qualitative descriptive analysis.

### Stage 3: field testing the revised PtDA prototype

Field testing by end users is a critical component of PtDA development as identified by IPDAS [19]. As opposed to the alpha testing, field testing solicits feedback on the PtDA from patients within the typical clinical setting. The primary outcome of the field testing is to evaluate the feasibility of using the PtDA at the point-of-care and to guide any final revisions, with secondary outcomes including acceptability, and potential of the PtDA to have the desired impact without adverse consequence.

We will perform a field test of the revised paper-based, pre-consultation PtDA prototype resulting from the alpha testing phase using a pre-/post-test study design and embedded qualitative feedback. A pre/post-test design has been chosen as it allows for evaluation of the secondary outcomes of the PtDA having the desired impact, which will provide us with baseline data to calculate a sample size for a larger evaluation study [47]. The prototype will be given to patients following initial presentation with the injury in the emergency department and self-administered by the patient at home prior to final treatment consultation with the orthopaedic surgeon.

#### Participants

Patient recruitment will take place at The Ottawa Hospital Emergency Department (ED). The Ottawa Hospital serves a population of 1.2 million people for orthopaedic care in Eastern Ontario, Canada and sees an average of 60 acute Achilles tendon rupture annually, or an average of 5 per month.

Eligibility for participation will include (1) adult patients over 18 years, (2) clinical or radiographically confirmed Achilles tendon rupture, (3) presentation and immobilization within 72 h of injury, (4) willingness to consent to the study, and (5) ability to speak and read English. Patients will be excluded for (1) re-rupture of the Achilles tendon, (2) musculotendinous junction tears and (3) delayed presentation over 72 h as this limits the ability to optimally treat with non-surgical methods.

#### Procedure

**Fig. 3:**
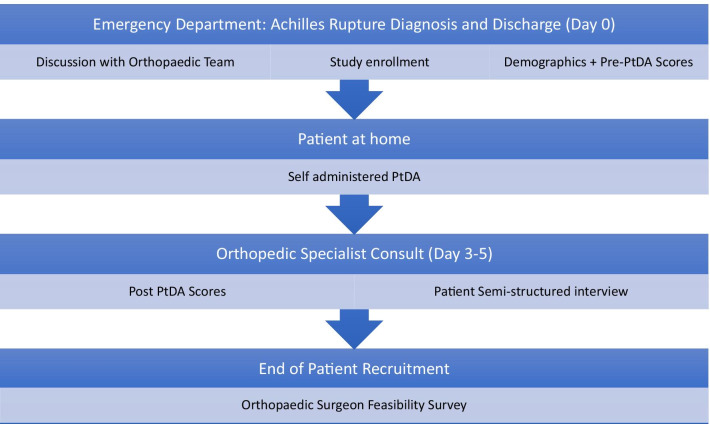
Patient participant study procedure

The procedure flow for patient recruitment is summarized in Fig. 3. Upon consultation from the emergency department physician, a member of the orthopaedic team (resident, staff physician or physician assistant) will meet patients presenting with an acute Achilles tendon rupture. The orthopaedic team will confirm the diagnosis, discuss the diagnosis and management options with the patient. Concurrently, they will verify if the patient meets inclusion criteria, discuss the study procedures, answer patients’ questions, and obtain the patients’ signed consent to participate. The patient will be asked to complete a baseline questionnaire including questions about their demographics (age, sex, gender, occupation, highest level of education) and secondary outcome measures. The patient will be splinted in plantarflexion in keeping with standard practice and given a copy of the PtDA to review and complete in preparation for their follow-up appointment in the orthopaedic clinic.

As per usual clinical practice, patients will be given a follow-up appointment in the orthopaedic plaster room 3–5 days following their emergency department visit. At the plaster room, the orthopaedic resident or surgeon will discuss the treatment options, answer patients’ questions and make the treatment decision with the patient. Either immediately following or within 2 weeks, the research assistant will ask the patient to complete a questionnaire including the secondary outcome measures and conduct the semi-structured interview.

We will also obtain feedback from orthopaedic residents and surgeons who consulted with patients in the study to determine the clinician experience with patients using the PtDA. After the last patient is recruited, these orthopaedic surgeons will be sent a link to an online survey using the SurveyMonkey (San Mateo CA) platform to elicit feedback on the PtDA.

#### Outcomes and instruments

Field test outcomes of interest have been chosen based on those recommended by IPDAS [18]. Field test outcomes are summarized in Table 1 and include: (1) feasibility of PtDA administration and use by patients, (2) barriers to PtDA use among patients and clinicians, (3) patients’ acceptability of the PtDA, and (4) potential of the PtDA to have the desired impact without adverse consequence.

**Table 1:** Summary of field test outcome measures

Outcome	Instruments	Measure of Success
1. Feasibility		
a. Patients		
	Metrics of use	At least 80% recruitment
		At least 80% PtDA completion
		At least 80% data completion
b. Surgeons	Patient semi-structured interview	Identifying resolvable barriers to PtDA use
	Metrics of use	At least 80% use for eligible patients
	Surgeon survey	Identifying resolvable barriers to PtDA use
2. Acceptability		
Amount of information	Post-PtDA	At least 66% perceive as acceptable
Length	Patient acceptability questionnaire	
Clarity		
Balance of presentation		
3. Potential for PtDA to have desired effect without adverse consequence		
	Post-PtDA	
	Patient preparation for decision making scale (PDMS)	Score of at least 66/100
	Practitioner preparation for decision making scale (PDMS)	Score of at least 66/100
	Pre- and post-PtDA	
	Knowledge test	At least 66% demonstrate improvement from pre to post PtDA administration
	SURE test	66% demonstrate improvement from pre to post PtDA administration
		And/or:
		> 66% Score 4/4 on post PtDA SURE test

Specific patient feasibility metrics will include the (1) percent of screened eligible patients recruited, (2) reasons for ineligibility, (3) percentage of recruited patients that completed the PtDA prior to the follow-up appointment and the (4) percentage of missing data on follow up outcome questionnaires. We will set a level of success at 80% participant recruitment of those meeting eligibility, 80% participant completion of the PtDA, and 80% minimal completed data on follow up questionnaires, in keeping with levels set in previously performed field tests [48].

The semi-structured interview with the patients will ask them to share their experiences on using the PtDA, their experiences discussing the decision with the orthopaedic surgeon, suggestions to improve the process of using the PtDA, formatting, and any barriers to use.

Acceptability of the PtDA, will be evaluated using the OAT[49] previously used in the alpha test phase. This validated instrument measures key outcomes including; amount of information, length, clarity and balanced presentation, which is important for avoiding biased presentation of information on options.

To evaluate whether the PtDA potential to have the desired impact without adverse consequence, patients will complete a knowledge test[50] and the SURE test[31] as a pre-/post-outcome and the Preparation for Decision Making Scale (PDMS) [51] as a Post-PtDA outcome measure.

Patient knowledge will be measured using a knowledge test developed by the steering group and embedded within the PtDA. The test will be adapted from the template available on the Ottawa Decision Support Group website [50] and will consist of 4–6 questions about key content important to know when making this decision. This format has been adapted for several clinical conditions and been shown to have high internal consistency and validity. Patient knowledge of their specific pathology and treatment options underlies one of the pillars of informed consent [52], and improved decision quality is based on patients making an informed, value-based decision [13].

The SURE test will be used to screen patients for decisional conflict [31]. It has four questions with ‘yes’ or ‘no’ responses in the domains of certainty, knowledge, values and support. Any ‘no’ answer is indicative of clinically significant decisional conflict. This test has been validated in a range of clinical settings [53, 54].

We will use the Preparation for Decision Making Scale (PDMS) [51] to measure patients’ perceived usefulness of a PtDA in preparing for a medical consultation. This specifically measures the IPDAS criteria for the quality of the decision process. This instrument consists of 10 questions to be ranked on a 5-point Likert scale, has both high reliability and internal consistency with a Cronbach’s alpha of 0.92–0.96 [55].

The survey of orthopaedic surgeons will ask questions about how the PtDA influenced the patient-surgeon encounter. Data collected will aim to quantify experience with the PtDA including (1) number of times and reasons the PtDA was used or not used for eligible patients, (2) if they plan to continue to use the PtDA and (3) barriers and facilitators to implementation in usual clinical care. Specific feedback will be sought with respect on how and when the PtDA can best be administered in the patient journey to optimize uptake by both clinicians and patients. As part of the survey, we will also use the 11-question practitioner version of the PDMS to evaluate clinician views on how effective the PtDA was on the patient encounter [51].

#### Sample size

There is a paucity of guidance for sample size justification for field test methods. With the primary objectives being feasibility of use in the clinical setting, we will aim to recruit a sample of 30 patient participants. This sample size is consistent with minimum numbers required to demonstrate adequate feasibility in pilot studies [56], and consistent with other pre-/post-studies testing PtDAs with patients facing the decision [57–59]. Based on usual referral volumes, we expect to recruit 3 patients per month and complete study recruitment after 10–12 months.

#### Data management and analysis

Quantitative data will be uploaded and stored in an encrypted Microsoft Excel spreadsheet. As the focus of the field test is on feasibility rather than efficacy outcomes, analysis will be primarily descriptive with baseline participant characteristics and feasibility data presented using frequencies and percentages. Tests for data normality will be performed and summarized using means and standard deviation if normally distributed, and medians and ranges if not. For post-test only outcomes (acceptability, PDMS), patients’ responses will be summarized and described. For pre-/post-test outcomes (knowledge and SURE test), questionnaires will be compared using the student t-test if normally distributed and using the Wilcoxon rank-sum test if not. Data will be disaggregated by sex and gender to examine how these variables may affect patient and clinician experience with the PtDA and associated outcome measures. Qualitative data from the semi-structured interviews will be transcribed verbatim and analyzed using thematic analysis as described for alpha testing.

There are no known defined standards for determining success of field testing measures. As such, we have determined the following a-priori criteria as determinants for success of the field test: (1) no insurmountable barriers identified from semi-structured interviews or open-ended feedback based on review from the steering group, (2) minimum of 80% targets for feasibility outcomes, (3) minimum of 66% of participants find the PtDA acceptable and balanced, (4) minimum score of 66 on the PDMS (5) 66% demonstrate nominal improvement on pre-/post-test outcomes for desired impact (knowledge and SURE). For the SURE test, an overall finding of > 66% participants responding with a 4/4 score will also be deemed a measure of success. If the cutoffs for determining success of the field test are not reached, revisions to the PtDA will be discussed and implemented by the steering group with further recruitment and testing at the discretion of the steering group.

## Discussion

This is a multi-faceted study protocol outlining the development, alpha and field testing of a pre-consultation PtDA for patients presenting with an acute Achilles tendon rupture. The need for a decision support tool for this treatment decision is evident from the existence of longstanding controversy in the orthopaedic community about the effectiveness of surgery versus conservative management [26, 27] and regarding important differences in the benefit-to-harm profile offered by the varying treatment methods. The treatment decision for patients with acute Achilles tendon rupture is time-sensitive, and so feasibility testing with patients presenting to the emergency department with these injuries is imperative to ensure the PtDA can be effectively administered in a timely fashion.

### Strengths and limitations

A major strength of this protocol lies in the use of an iKT approach to develop the PtDA. This approach has been demonstrated to maximize knowledge users input, increase use, and improve impact [22]. We have adhered to a rigorous methodology guided by the ODSF and the IPDAS to ensure the PtDA is developed to a high quality standard [60]. Additionally, engaging stakeholders from multiple disciplines and Canadian locations in the development and alpha testing of the PtDA will ensure the end product is usable and optimized to meet the needs of a variety of patients and clinicians. This approach will also raise early awareness of the PtDA’s existence, necessary for achieving our of aim of public availability and national dissemination. Additionally, the inclusion of qualitative methods allows for collection of deeper stakeholder insights, perceptions and opinions [61], all of which are critical in designing a PtDA that will optimize user uptake.

Limitations of this study protocol include the arbitrary success endpoints for both alpha and field testing outcomes. To our knowledge, discrete success endpoints have not been previously described. Our endpoints remain largely qualitative and at the discretion of the steering committee. Despite this, we are using methods recommended by IPDAS [19], and will have steering committee members with expertise in PtDA development and evaluation. Further work and study to better define these endpoints is necessary. Additionally, the sample is unlikely to allow us to identify sex and gender-specific differences from either patients or clinicians because it will likely be majority male, reflecting the populations represented; Achilles tendon ruptures occur far more frequently in men [2], and orthopaedic surgeons are disproportionately male. Nonetheless, our analysis plan details an effort to capture and analyze these differences. Finally, there are inherent limitations of the before/after study design, including the lack of a control arm. This makes it difficult to control for confounding factors that may affect patient outcomes, such as independent research into treatment options. However, the design been previously used in the evaluating PtDAs [47] and offers the advantage of facilitating patient recruitment.

## Conclusions

Patient decision aids are meant to improve patient preparation for decision-making. By providing foundational information and encouraging patients to reflect on relevant personal values for outcomes of options, these tools may facilitate the patient-physician decision making interaction and enhance patient-centered care. Our aim is to develop, alpha test and field test this pre-consultation Achilles tendon rupture decision aid. We hope this will be the first of a series of orthopaedic PtDAs developed as part of a quality improvement initiative for facilitating shared decision making for patients receiving musculoskeletal care.

## Supplementary Information

**Additional file 1: Appendix A**. Ottawa Acceptability Tool (Patient Version): supplementary material; PtDA acceptability to patients will be evaluated using this questionnaire.

**Additional file 2: Appendix B**. Ottawa Acceptability Tool (Clinician Version): supplementary material; PtDA acceptability to clinicians will be evaluated using this questionnaire.

**Additional file 3: Appendix C**. System Usability Scale: supplementary material; PtDA usability will be evaluated using this questionnaire.

## Data Availability

The datasets used and/or analyzed during the current study are available from the corresponding author on reasonable request.

## Abbreviations

iKT: Integrated knowledge translation
IPDAS: International patient decision aids standards
OAT: Ottawa acceptability tool
ODSF: Ottawa decision support framework
PtDA: Patient decision-aid
PDMS: Preparation for decision making scale
SSI: Semi-structured interviews
SDM: Shared decision-making
SUS: System usability scale
