# Supplementary material for: Development and field testing of a patient decision aid for management of acute Achilles tendon rupture: a study protocol
Source: BMC Med Inform Decis Mak. 2021 Jul 24;21:225. doi: 10.1186/s12911-021-01589-5 (PMC8310595; doi:10.1186/s12911-021-01589-5)
Supplement: Supplementary file 1 — Additional file 1: Appendix A. Ottawa Acceptability Tool (Patient Version): supplementary material; PtDA acceptability to patients will be evaluated using this questionnaire. [file 12911_2021_1589_MOESM1_ESM.docx]

| **Ottawa Acceptability Tool (Patient Version)** | | | | | | | | | | |
| --- | --- | --- | --- | --- | --- | --- | --- | --- | --- | --- |
|  |  | | | | | | | | | |
| **My thoughts on the education package on Achilles Tendon Rupture Management** | | | | | | | | | | |
| We would like to know what you think about the education package you have just received. | | | | | | | | | | |
|  | | | | | | | | | | |
| **1.** | | Please rate each section, by circling ‘poor’, ‘fair’, ‘good’, or ‘excellent’ to show what you think about the way the information was presented on: | | | | | | | | |
|  | |  | |  | |  | |  | |  |
|  | | Impact of an Achilles Tendon Rupture | | Poor | | Fair | | Good | | Excellent |
|  | |  | |  | |  | |  | |  |
|  | | Risk Factors for Complications | | Poor | | Fair | | Good | | Excellent |
|  | |  | |  | |  | |  | |  |
|  | | Types of Research Studies | | Poor | | Fair | | Good | | Excellent |
|  | |  | |  | |  | |  | |  |
|  | | Non-Surgical Options | | Poor | | Fair | | Good | | Excellent |
|  | |  | |  | |  | |  | |  |
|  | | Evidence About Surgery | | Poor | | Fair | | Good | | Excellent |
|  | |  | |  | |  | |  | |  |
|  | | Stories About Others | | Poor | | Fair | | Good | | Excellent |
|  | |  | |  | |  | |  | |  |
|  | |  | |  | |  | |  | |  |
| **2.** | | The length of presentation was *(check one)* | | | | | | | | |
|  | | 🞏 | Too long | | | | | | | |
|  | | 🞏 | Too short | | | | | | | |
|  | | 🞏 | Just right | | | | | | | |
|  | |  | |  |  | |  | |  | |
| **3.** | | The amount of information was *(check one)* | | | | | | | | |
|  | | 🞏 | Too much information | | | | | | | |
|  | | 🞏 | Too little information | | | | | | | |
|  | | 🞏 | Just right | | | | | | | |
|  | |  |  | | | | | | | |
| **4.** | | I found the presentation *(check one)* | | | | | | | | |
|  | | 🞏 | Slanted towards choosing non-operative treatment | | | | | | | |
|  | | 🞏 | Slanted towards choosing surgery | | | | | | | |
|  | | 🞏 | Balanced | | | | | | | |
|  | |  |  | | | | | | | |

| **5.** | Would you have found this decision aid useful when you were making your decision about treatment for your Achilles tendon rupture? | |
| --- | --- | --- |
|  | 🞏 | Yes |
|  | 🞏 | No |
|  | Comments: | |
|  |  | |
|  |  | |
|  |  | |
| **6.** | What did you think of the rest of the personal worksheet? Did it make the decision | |
|  | 🞏 | Easy |
|  | 🞏 | More difficult |
|  | Comments: | |
|  |  | |
|  |  | |
|  |  | |
| **7.** | Do you think we included enough information to help a patient decide on treatment for Achilles tendon rupture? | |
|  | 🞏 | Yes |
|  | 🞏 | No |
|  | Comments: | |
|  |  | |
|  |  | |
|  |  | |
| **8.** | What did you like about the decision aid and worksheet? | |
|  |  | |
|  |  | |
|  |  | |
|  |  | |
|  |  | |
| **9.** | What suggestions do you have to improve the decision aid or worksheet? | |
|  |  | |
|  |  | |
|  |  | |
|  |  | |
|  |  | |
