# Supplementary material for: Development and field testing of a patient decision aid for management of acute Achilles tendon rupture: a study protocol
Source: BMC Med Inform Decis Mak. 2021 Jul 24;21:225. doi: 10.1186/s12911-021-01589-5 (PMC8310595; doi:10.1186/s12911-021-01589-5)
Supplement: Supplementary file 2 — Additional file 2: Appendix B. Ottawa Acceptability Tool (Clinician Version): supplementary material; PtDA acceptability to clinicians will be evaluated using this questionnaire. [file 12911_2021_1589_MOESM2_ESM.docx]

| **Ottawa Acceptability Tool (Clinician Version)** | | | | | | |
| --- | --- | --- | --- | --- | --- | --- |
|  |  | | | | | |
| **My thoughts on the education package on Achilles Tendon Rupture Management** | | | | | | |
| The following set of questions asks about your perceptions of the decision support strategy you have been assigned. We are interested in your reactions to the strategy so please try to answer these questions in the same way you would if you had not agreed to take part in this study and were seeing the strategy for the first time. Please indicate how strongly you agree or disagree with each statement by *circling* the appropriate number. | | | | | | |
|  | | | | | | |
|  |  | | | | | |
| In General: | | **Strongly Disagree** |  | **Neutral** |  | **Strongly Agree** |
| It will be easy for me to use with my patients | | 1 | 2 | 3 | 4 | 5 |
| It is easy for me to understand how it can be used with my patients | | 1 | 2 | 3 | 4 | 5 |
| It will be easy for me to experiment with using the strategy before making a final decision to adopt it. | | 1 | 2 | 3 | 4 | 5 |
| The results of using the strategy will be easy to see. | | 1 | 2 | 3 | 4 | 5 |
| This strategy is better than how I usually go about helping patients decide about Achilles tendon rupture management. | | 1 | 2 | 3 | 4 | 5 |
| This strategy is compatible with the way I think things should be done. | | 1 | 2 | 3 | 4 | 5 |
| The use of this strategy is a more cost-effective than my usual approach to helping patients decide about Achilles tendon rupture management | | 1 | 2 | 3 | 4 | 5 |
| Compared with my usual approach, this strategy will result in my patients making more informed decisions. | | 1 | 2 | 3 | 4 | 5 |
| Using this strategy will save me time. | | 1 | 2 | 3 | 4 | 5 |
| This strategy is a reliable method of helping patients make decisions about Achilles tendon rupture management | | 1 | 2 | 3 | 4 | 5 |
| Pieces or components of the strategy can be used by themselves. | | 1 | 2 | 3 | 4 | 5 |
| This type of strategy is suitable for helping patients make value laden choices. | | 1 | 2 | 3 | 4 | 5 |
| This strategy complements my usual approach. | | 1 | 2 | 3 | 4 | 5 |
| Using this strategy does not involve making major changes to the way I usually do things | | 1 | 2 | 3 | 4 | 5 |
| There is a high probability that using this strategy may cause/result in more benefit than harm | | 1 | 2 | 3 | 4 | 5 |
|  |  | | | | | |
