# Supplementary material for: Development and field testing of a patient decision aid for management of acute Achilles tendon rupture: a study protocol
Source: BMC Med Inform Decis Mak. 2021 Jul 24;21:225. doi: 10.1186/s12911-021-01589-5 (PMC8310595; doi:10.1186/s12911-021-01589-5)
Supplement: Supplementary file 3 — Additional file 3: Appendix C. System Usability Scale: supplementary material; PtDA usability will be evaluated using this questionnaire. [file 12911_2021_1589_MOESM3_ESM.docx]

| **System Usability Scale** | | | | | | |
| --- | --- | --- | --- | --- | --- | --- |
|  |  | | | | | |
|  | | **Strongly Disagree** |  | **Neutral** |  | **Strongly Agree** |
| I think that I would like to use this tool. | | 1 | 2 | 3 | 4 | 5 |
| I found the tool unnecessarily complex. | | 1 | 2 | 3 | 4 | 5 |
| I think that I would need support to be able to use this system. | | 1 | 2 | 3 | 4 | 5 |
| I found the various parts in this tool were well integrated. | | 1 | 2 | 3 | 4 | 5 |
| I thought there was too much inconsistency in this tool. | | 1 | 2 | 3 | 4 | 5 |
| I would imagine that most people would learn to use this tool very quickly. | | 1 | 2 | 3 | 4 | 5 |
| I found the tool very cumbersome to use. | | 1 | 2 | 3 | 4 | 5 |
| I felt very confident using the tool. | | 1 | 2 | 3 | 4 | 5 |
| I needed to learn a lot of things before I could get going with this tool. | | 1 | 2 | 3 | 4 | 5 |
|  |  | | | | | |
